# Enhancing recurrence risk prediction for bladder cancer using multi-sequence MRI radiomics

**DOI:** 10.1186/s13244-024-01662-3

**Published:** 2024-03-25

**Authors:** Guoqiang Yang, Jingjing Bai, Min Hao, Lu Zhang, Zhichang Fan, Xiaochun Wang

**Affiliations:** 1 Department of Radiology, the First Hospital of Shanxi Medical University, Taiyuan, Shanxi China; 2 College of Medical Imaging, Shanxi Medical University, Taiyuan, Shanxi China

**Keywords:** Bladder cancer, MRI, Radiomics, Preoperative nomogram, Recurrence

## Abstract

**Objective:**

We aimed to develop a radiomics-clinical nomogram using multi-sequence MRI to predict recurrence-free survival (RFS) in bladder cancer (BCa) patients and assess its superiority over clinical models.

**Methods:**

A retrospective cohort of 229 BCa patients with preoperative multi-sequence MRI was divided into a training set (*n* = 160) and a validation set (*n* = 69). Radiomics features were extracted from T2-weighted images, diffusion-weighted imaging, apparent diffusion coefficient, and dynamic contrast-enhanced images. Effective features were identified using the least absolute shrinkage and selection operator (LASSO) method. Clinical risk factors were determined via univariate and multivariate Cox analysis, leading to the creation of a radiomics-clinical nomogram. Kaplan-Meier analysis and log-rank tests assessed the relationship between radiomics features and RFS. We calculated the net reclassification improvement (NRI) to evaluate the added value of the radiomics signature and used decision curve analysis (DCA) to assess the nomogram’s clinical validity.

**Results:**

Radiomics features significantly correlated with RFS (log-rank *p* < 0.001) and were independent of clinical factors (*p* < 0.001). The combined model, incorporating radiomics features and clinical data, demonstrated the best prognostic value, with C-index values of 0.853 in the training set and 0.832 in the validation set. Compared to the clinical model, the radiomics-clinical nomogram exhibited superior calibration and classification (NRI: 0.6768, 95% CI: 0.5549-0.7987, *p* < 0.001).

**Conclusion:**

The radiomics-clinical nomogram, based on multi-sequence MRI, effectively assesses the BCa recurrence risk. It outperforms both the radiomics model and the clinical model in predicting BCa recurrence risk.

**Critical relevance statement:**

The radiomics-clinical nomogram, utilizing multi-sequence MRI, holds promise for predicting bladder cancer recurrence, enhancing individualized clinical treatment, and performing tumor surveillance.

**Key points:**

• Radiomics plays a vital role in predicting bladder cancer recurrence.

• Precise prediction of tumor recurrence risk is crucial for clinical management.

• MRI-based radiomics models excel in predicting bladder cancer recurrence.

**Graphical Abstract:**

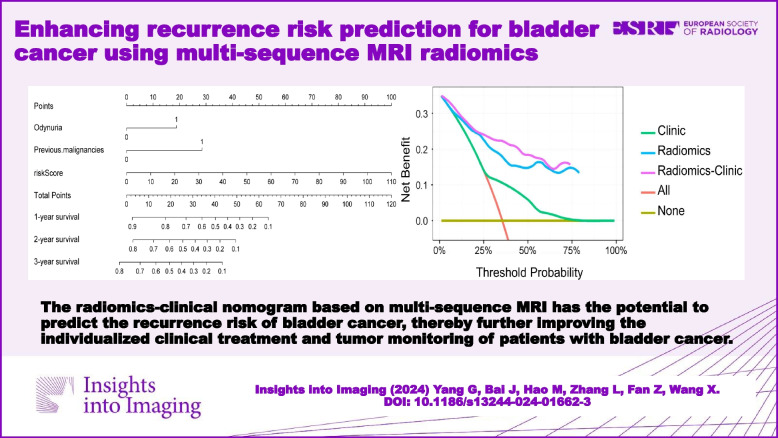

## Introduction

Bladder cancer (BCa) is the tenth most common cancer worldwide and is associated with high morbidity and mortality, especially in men. In 2020, 573,278 new cases and 212,536 deaths were reported. The reported incidence rate was 9.5 per 100,000, and the mortality rate was 3.3 per 100,000 [1, 2]. BCa has become a great challenge in oncology due to the need for long-term surveillance and invasive treatment. Distinguished into non-muscle-invasive bladder cancer (NMIBC, ≤ T1 stage) and muscle-invasive bladder cancer (MIBC, ≥ T2 stage), BCa’s diverse molecular characteristics and clinical outcomes necessitate distinct treatment approaches [3, 4]. For early-stage NMIBC, the recommended course involves transurethral resection of bladder tumor (TURBT) supplemented by Bacille Calmette-Guérin (BCG) therapy [5]. However, patients facing more advanced MIBC confront bleak prognoses, typically requiring radical cystectomy (RC) followed by bilateral pelvic lymph node dissection (PLND) [6].

Recurrence of BCa remains a concern, particularly for NMIBC patients, where recurrence rates are as high as 61% within 2 years of TURBT [7, 8]. MIBC patients are not spared either, as the metastatic or recurrence rate ranges from 5 to 50% within the first 2 years after RC [4]. Inadequate treatment for those at high risk of recurrence can have fatal consequences, underscoring the imperative of robust, long-term monitoring [9]. Thus, the need arises for the development of more effective early treatment strategies and closer follow-up management to enhance patient survival [10].

The management of BCa often falters, and conventional clinical staging systems have proven unreliable in predicting prognosis and guiding treatment decisions. The predominant risk assessment methods, such as the European Organization for Research and Treatment of Cancer (EORTC) risk model [8] and the Spanish Club of Urology Oncology Treatment (CUETO) scoring model [11], rely on clinical and histological factors to assess risk of recurrence and progression. Regrettably, both models exhibit poor discriminatory power for recurrence and consistently overestimate the risk for high-risk patients [12]. This not only jeopardizes the patient’s physical well-being but also compounds the complexity of management [4, 13, 14].

To address these shortcomings, a novel concept in biomarker assessment known as “radiomics” has emerged, showing substantial promise in BCa prognosis and follow-up management [15, 16]. Radiomics leverages medical imaging to analyze the relationship between phenotypic image features (radiomics biomarkers) and tumor diagnosis, thereby refining patient risk stratification and treatment decisions. Among the array of imaging modalities, MRI shines with its ability to offer multi-sequence imaging, high-resolution soft tissue visualization, and multi-level structural and functional information [17–19]. When combined with the Vesical Imaging Reporting and Data System (VI-RADS), MRI facilitates a comprehensive assessment of image classification and muscular infiltration [20–22]. Several studies have demonstrated the efficacy of radiomics based on CT or MRI in accurately predicting BCa’s preoperative grading, lymph node metastasis, and myometrial invasion status (MIS), thereby guiding clinical treatment and prognosis assessment [23, 24].

Yet, a research gap remains regarding radiomics signatures based on multi-sequence MRI for evaluating BCa recurrence risk. A previous study [25] found that radiology characteristics based on diffusion-weighted imaging (DWI) could independently predict progression-free survival (PFS) in MIBC patients. An opportunity exists for further research regarding the additional value of crafting a nomogram incorporating multi-sequence MRI features alongside clinical risk factors in a collective study.

In this study, we embark on developing a radiomics nomogram to forecast BCa patient prognosis. Our nomogram draws from radiomics features extracted from multi-sequence MRI, including T2-weighted image (T2WI), DWI, apparent diffusion coefficient (ADC), and dynamic contrast-enhanced (DCE) imaging, to predict recurrence-free survival (RFS) in BCa patients. We also investigated a combined model by merging clinical risk factors with radiomics features, to demonstrate the enhanced value of such an integrated approach in stratifying BCa recurrence risk.

## Materials and methods

### Patients

This retrospective study was approved by the Institutional Review Board of the First Hospital of Shanxi Medical University. Written informed consent was obtained from all patients in this study. We conducted a retrospective search within the pathology and radiology databases of the First Hospital of Shanxi Medical University, spanning from January 2018 to June 2021, to identify patients diagnosed with BCa. Subsequently, we analyzed the clinical data of 229 patients with pathologically confirmed BCa.

**Fig. 1:**
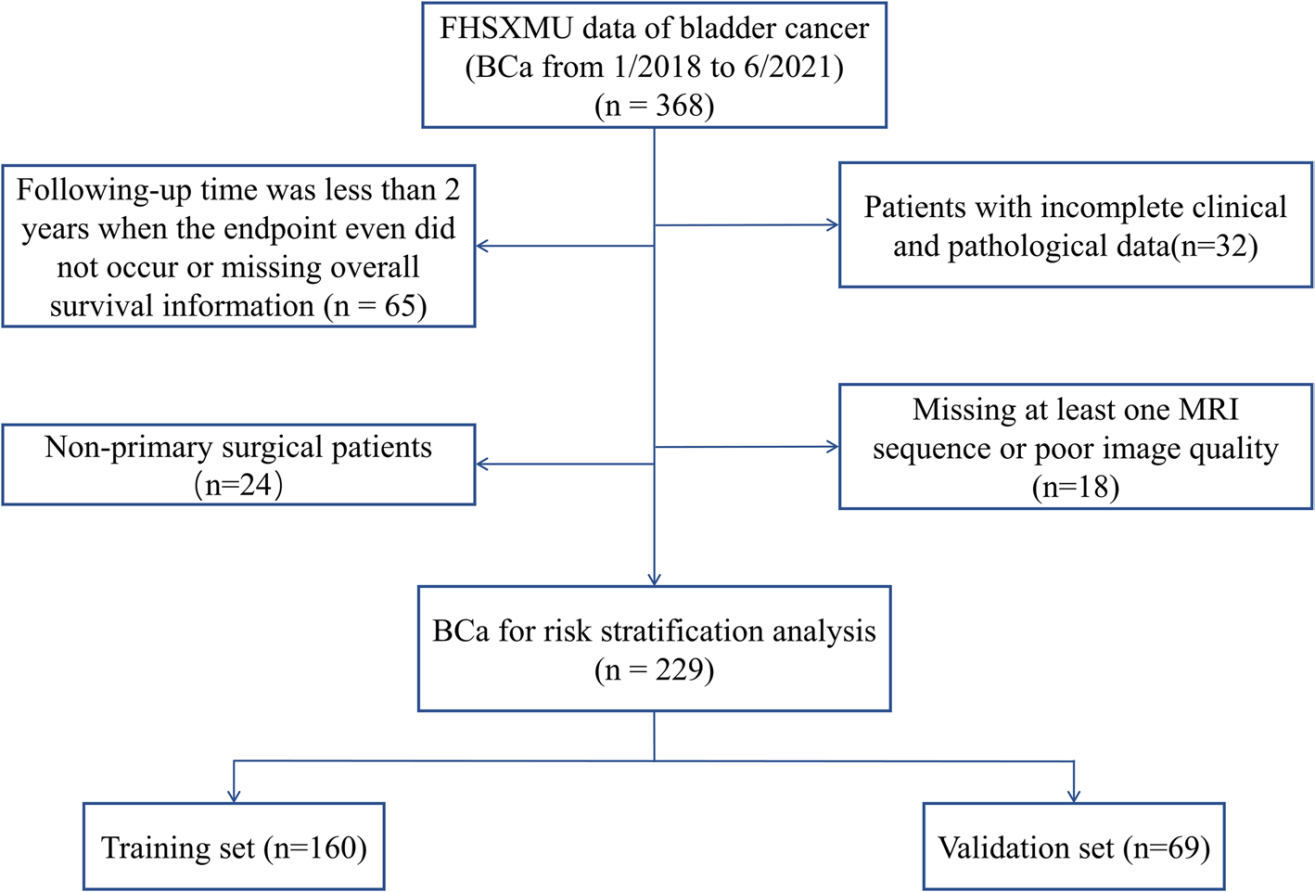
Flowchart shows selection criteria for the 229 patients in the study group

Inclusion criteria were defined as follows: (1) confirmation of BCa through pathology following TURBT or RC; (2) initial TURBT or cystectomy procedure; (3) MRI examination conducted within 3 weeks prior to surgery; and (4) the availability of complete clinical and pathological data. Exclusion criteria comprised the following: (1) receipt of preoperative radiotherapy or chemotherapy; (2) cases exhibiting poor image quality or tumors with a diameter less than 3 mm, making delineation unfeasible; (3) instances with incomplete clinical and pathological data; or (4) patients lost to follow-up or follow-up was less than 2 years. The process of case inclusion and screening is presented in Fig. 1.

### Clinical data collection and patient follow-up

We collected clinical data from 229 patients with confirmed BCa, including seven parts: demographic characteristics (such as age at diagnosis, BMI, gender, smoking, and drinking), clinical characteristics (such as frequent urination, urinary urgency, odynuria, urinary incontinence, low back pain, and previous malignancies), serum laboratory information (such as total cholesterol, triglyceride, high-density lipoprotein, low-density lipoprotein, urea, creatinine, and uric acid), tumor characteristics (such as tumor location, tumor size, and tumor number), pathological data (such as pathological grading, and MIS), treatment information (such as infusion drug, and surgical methods), and survival information (RFS).

Following surgical intervention, each patient underwent a meticulously planned follow-up regimen, involving an initial assessment within 3–5 months post-surgery, subsequent evaluations every 6 months for a duration of 2 years, and annual appointments thereafter. These follow-up assessments entailed comprehensive cystoscopy and imaging examinations (CT or MRI) to scrutinize for any signs of suspected bladder tumor recurrence. For the purpose of this study, recurrent tumors were defined as tumors that reappeared within the bladder, prostate, urethra, pelvis, or ileum subsequent to surgical intervention. By the end of follow-up, 81 BCa patients experienced a recurrence. Importantly, we documented the time to RFS for each patient, calculated from the date of their initial surgery.

### MRI protocol

All MRI scans were conducted using a 3.0-T MRI scanner (Skyra: Siemens, Erlangen, Germany) equipped with an 8-channel truncal phased-array coil or body coil. Specific scan range, scan sequence, and detailed scan parameters are provided in Additional file 1.

### Tumor ROI segmentation

Two experienced radiologists, referred to as Reader1 and Reader2, each possessing over 8 years of experience working with MRI, manually delineated the entire bladder tumor using ITK-SNAP 3.8.0 (http://www.itk-snap.org/). Importantly, they remained blinded to the patient’s pathological findings throughout the procedure. Subsequently, the axial images from T2WI, DWI, ADC, and DCE were segmented to extract the volume of interest (VOI). In cases where disagreements arose, a resolution was reached through consultation.

Additionally, to assess the consistency of the image feature delineation both within and between observers, 1 month later, the VOI of 15 recurrent and 15 non-recurrent patients were randomly selected and delineated by a single radiologist, Reader1. This allowed for the evaluation of the intraclass correlation coefficient (ICC) for the delineated image features.

**Fig. 2:**
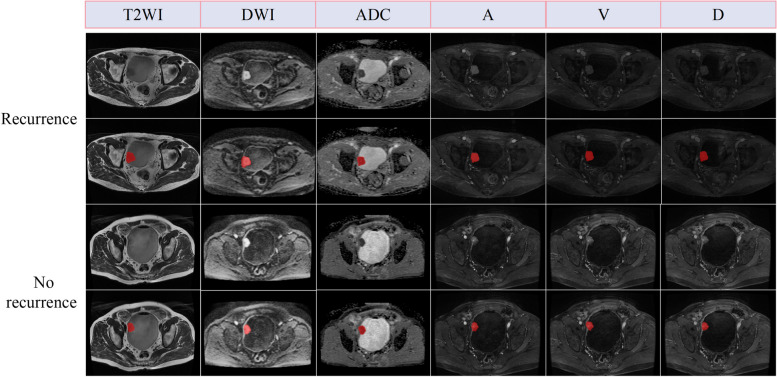
A 69-year-old male patient presented with recurrent BCa on follow-up surveillance and an 85-year-old male patient with follow-up surveillance for non-recurrent BCa

The tumor delineation contours of all sequences are shown in Fig. 2. The definition of tumor margin on different weighted images needs to be determined based on the signal characteristics. The specific rules for this process can be found in Additional file 2.

### Radiomics feature extraction

We used the open-source software FAE (https://github.com/salan668/FAE) using PyRadiomics package to extract radiomics features from T2WI, DWI, ADC, and DCE images of BCa patient. The VOI delineated on T2WI, DWI, ADC, and DCE images for each BCa patient underwent a series of preprocessing steps. The radiomics feature extraction steps and feature categories are described in Additional file 3.

### Development of the radiomics nomogram

Firstly, the reproducibility of each feature delineation process was quantified by evaluating the ICC values of the inter- and intra-group datasets, and the radiomics features with ICC < 0.75 were excluded for subsequent analysis. The remaining stable features were normalized and significant radiomics features were selected using univariate Cox regression with *p* < 0.05. Univariate analysis and LASSO regression algorithm were used to select the optimal feature subset. The RFS prediction model of BCa was constructed based on the optimal radiomics features, and the radiomics score (radscore) of each patient was calculated. The cutoff value for the high and low-risk groups were identified by the median radscore in the training set, and Kaplan-Meier analysis was used to assess the potential association between radscore and RFS, which was validated in the validation set.

### Development of the clinical nomogram

Univariate Cox regression analysis was utilized to assess the association between clinical features and the RFS in patients. After finding a statistically significant difference (*p* < 0.05) between the recurrent and the non-recurrent, we conducted a multivariate Cox regression analysis and then selected independent predictors of BCa recurrence with *p* < 0.05. Based on this, a clinical model was developed and used for validation.

### Development of the radiomics-clinical nomogram

By incorporating the independent risk factors identified in the clinical model and integrating them with the radscore derived from the radiomics model as covariates, we have devised a practical and clinically relevant radiomics-clinical nomogram. This nomogram serves as a valuable tool for predicting early BCa recurrence and individualized RFS. To evaluate the performance of the nomogram and its goodness of fit, we utilized metrics such as the C-index and calibration curve, both in the training set and the validation set. In order to evaluate the nomogram’s diagnostic capabilities, we employed the net reclassification improvement (NRI) and compared it against the radiomics model and clinical model. Lastly, we conducted decision curve analysis (DCA) for all three models, providing insights into the clinical validity and utility of our proposed tool.

### Statistical analysis

The statistical analyses for this study were conducted using R version 4.2.0 (https://www.r-project.org/) and SPSS 26.0 (http://www.spss.com.cn). The one-sample Shapiro-Wilk test was used to assess the normality of numerical variables. For normally distributed data, we presented results as mean ± standard deviation (M ± SD), while non-normally distributed data were represented as median (interquartile range (IQR), 25th and 75th percentiles). Two-sample *t*-test was used to compare normally distributed data between groups, and the Mann-Whitney *U* test was used to compare non-normally distributed data. The chi-square test was used to analyze categorical data.

To gauge the relationship between the radscore derived from the prediction model and the RFS status of patients, we employed Kaplan-Meier analysis and Cox regression analysis to compute early recurrence rates. Statistical significance was established at a threshold of *p* < 0.05. The C-index was utilized to evaluate the model’s accuracy in predicting recurrence stratification and RFS performance in two sets.

## Results

### Clinical characteristics of the patients

The demographic and tumor characteristics of the entire patient cohort are summarized in Table 1. The median age of the 229 patients included in the study was 66 years, comprising 199 males and 30 females, with the age range spanned from 26 to 88 years.

**Table 1:** Characteristics of BCa patients in the training set and validation set

Characteristics	Overall (*n* = 229)	Training set (*n* = 160)	Validation set (*n* = 69)	*p* values
Age (years)	66.54 ± 10.70	65.00 ± 11.14	66.80 ± 9.55	0.244
BMI (kg/m^2^)	23.94 (21.88–26.48)	23.90 (21.52–26.47)	24.44 (22.70–26.94)	0.271
Gender				0.403
Female	30 (13.1%)	19 (11.9%)	11 (15.9%)	
Male	199 (86.9%)	141 (88.1%)	58 (84.1%)	
Smoking				0.649
No	128 (55.9%)	91 (56.9%)	37 (53.6%)	
Yes	101 (44.1%)	69 (43.1%)	32 (46.4%)	
Drinking				0.805
No	175 (76.4%)	123 (76.9%)	52 (75.4%)	
Yes	54 (23.6%)	37 (23.1%)	17 (24.6%)	
Frequent urination				0.386
No	174 (76.0%)	119 (74.4%)	55 (79.7%)	
Yes	55 (24.0%)	41 (25.6%)	14 (20.3%)	
Urinary urgency				0.412
No	171 (74.7%)	117 (73.1%)	54 (78.3%)	
Yes	58 (25.3%)	43 (26.9%)	15 (21.7%)	
Odynuria				0.441
No	182 (79.5%)	125 (78.1%)	57 (82.6%)	
Yes	47 (20.5%)	35 (21.9%)	12 (17.4%)	
Urinary incontinence				0.102
No	215 (93.9%)	147 (91.9%)	68 (98.6%)	
Yes	14 (6.1%)	13 (8.1%)	1 (1.4%)	
Low back pain				0.705
No	210 (91.7%)	146 (91.2%)	64 (92.8%)	
Yes	19 (8.3%)	14 (8.8%)	5 (7.2%)	
Total cholesterol (mmol/L)	4.37 (3.65–5.04)	4.41 (3.62–5.09)	4.33 (3.70–4.82)	0.627
Triglyceride (mmol/L)	1.28 (0.98–1.78)	1.28 (0.95–1.76)	1.28 (1.01–1.86)	0.619
High-density lipoprotein (mmol/L)	1.06 (0.93–1.27)	1.05 (0.92–1.27)	1.07 (0.95–1.31)	0.653
Low-density lipoprotein (mmol/L)	2.81 (2.29–3.32)	2.86 (2.25–3.35)	2.79 (2.34-3.19)	0.682
Urea (mmol/L)	5.65 (4.65–6.68)	5.62 (4.53–6.67)	5.92 (4.76–6.83)	0.377
Creatinine (μmol/L)	72.10 (63.20–81.40)	72.90 (64.93–82.48)	69.10 (60.25–76.85)	0.050
Uric acid (μmol/L)	321.96 ± 79.93	326.98 ± 76.47	310.32 ± 86.89	0.148
Previous malignancies				0.991
No	214 (93.4%)	149 (93.1%)	65 (94.2%)	
Yes	15 (6.6%)	11 (6.9%)	4 (5.8%)	
Tumor location				0.861
Posterior wall	99 (43.2%)	71 (44.4%)	28 (40.6%)	
Side wall, top wall	107 (46.7%)	73 (45.6%)	34 (49.3%)	
Trigone, neck	23 (10.0%)	16 (10.0%)	7 (10.1%)	
Tumor size				0.998
≤ 3 cm	146 (63.8%)	102 (63.7%)	44 (63.8%)	
> 3 cm	83 (36.2%)	58 (36.3%)	25 (36.2%)	
Tumor number				0.222
Single	159 (69.4%)	115 (71.9%)	44 (63.8%)	
Multiple	70 (30.6%)	45 (28.1%)	25 (36.2%)	
Pathological grading				0.210
Low	115 (50.2%)	76 (47.5%)	39 (56.5%)	
High	114 (49.8%)	84 (52.5%)	30 (43.5%)	
MIS				0.348
NMIBC (stage ≤ T1)	149 (65.1%)	101 (63.1%)	48 (69.6%)	
MIBC (stage ≥ T2)	80 (34.9%)	59 (36.9%)	21 (30.4%)	
Infusion drug				0.869
Pirarubicin hydrochloride	76 (33.2%)	54 (33.8%)	22 (31.9%)	
Gemcitabine hydrochloride	84 (36.7%)	57 (35.6%)	27 (39.1%)	
Epirubicin	44 (19.2%)	30 (18.8%)	14 (20.3%)	
Other chemotherapy drugs	25 (10.9%)	19 (11.9%)	6 (8.7%)	
Surgical methods				0.741
TURBT	175 (76.4%)	120 (75.0%)	55 (79.7%)	
Partial RC	12 (5.2%)	9 (5.6%)	3 (4.3%)	
RC	42 (18.3%)	31 (19.4%)	11 (15.9%)	
RFS, days	1023 (662–1316)	1003 (597–1318)	1055 (853–1316)	0.196

*BCa* bladder cancer, *BMI* body mass index, *MIS* myometrial invasion status *TURBT* Transurethral resection of bladder tumor, *RC* Radical cystectomy, *RFS* Relapse-free survival

All patients were randomly divided into a training set (*n* = 160) and a validation set (*n* = 69) in a ratio of 7:3. Among these, 58 patients in the training set and 23 patients in the validation set experienced recurrence. The median RFS for all patients who relapsed was 391 days, with a range of 18 to 1461 days, while the median RFS for all non-relapsed patients was 1173 days, ranging from 333 to 1812 days. Importantly, there were no significant differences in demographic distribution and clinical characteristics between the training set and the validation set (*p* = 0.050 to 0.998).

### Development and validation of the radiomics nomogram

**Fig. 3:**
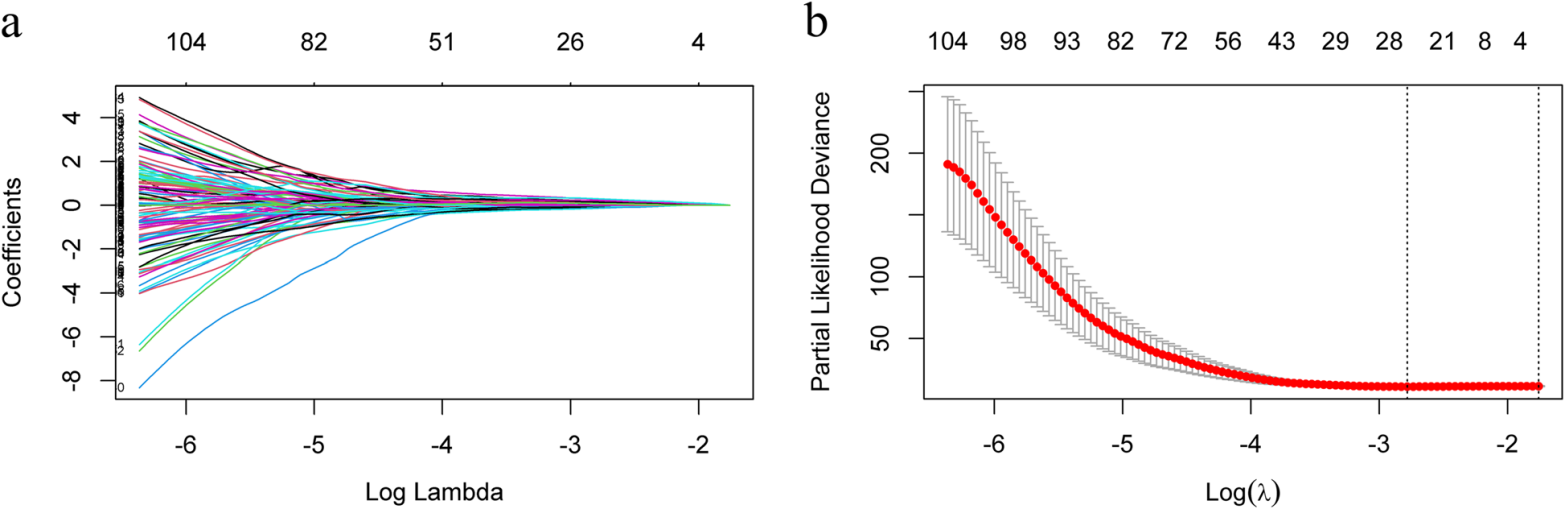
Dimension reduction of radiomics features in the LASSO Cox model

Following rigorous reliability testing (both intra-rater and inter-rater), we retained 7462 radiomics features, excluding those with an ICC < 0.75 from the initial 10686 features. Subsequently, through univariate Cox regression analysis, we further refined the features to 263. To mitigate overfitting and identify the optimal radiomics signature, we employed the LASSO technique within the Cox proportional hazards regression model (illustrated in Fig. 3). Ultimately, by identifying the minimum *λ* value, we identified 22 MRI radiomics features with non-zero coefficients, demonstrating a higher C-index in predicting BCa recurrence. Details on these specific characteristics, corresponding coefficients, and hazard ratios (HR) can be found in Additional Fig 1. The radiomics features were combined linearly to derive the radscore.

**Fig. 4:**
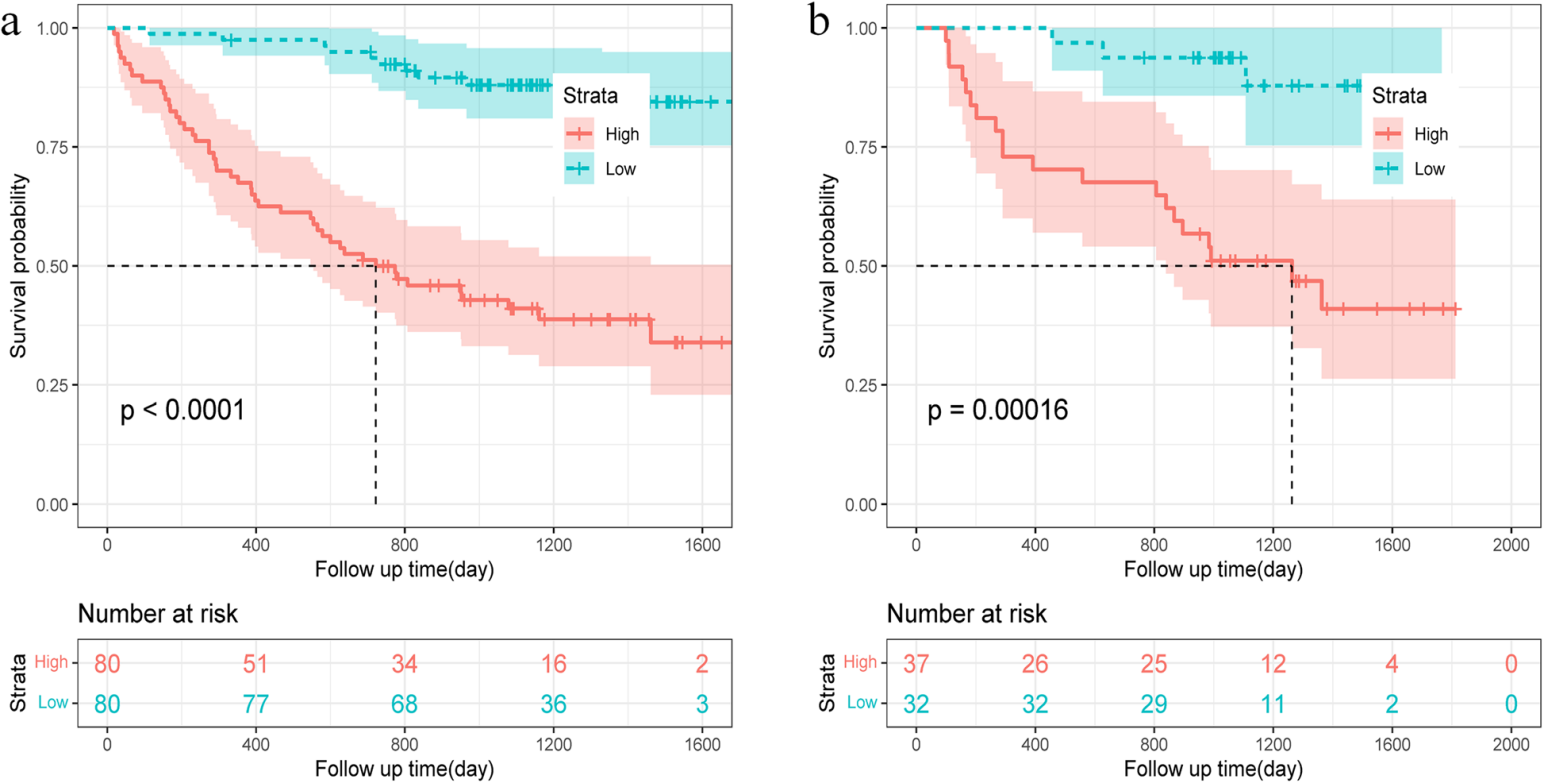
Kaplan-Meier analysis of RFS according to the radiomics signature in the training data set (**a**) and validation data set (**b**). The significant association of the radiomics signature with RFS was validated

The radiomics model’s recurrence prediction performance was assessed using the C-index, which had a score of 0.823 [95% confidence interval (CI), 0.794–0.852] for the training set and 0.811 (95% CI, 0.772–0.850) for the independent validation set. Each patient’s radscore was computed based on the radiomics features within the training dataset. The median radscore of the training set was 0.809 as the cut-off point, and the patients were divided into high-risk group and low-risk group. Kaplan-Meier curves of radiomics features in the training and validation sets are presented in Fig. 4a and 4b, respectively. In the training set, the radscore exhibited a significant association with RFS (*p* < 0.0001, HR = 1.044, 95% CI: 1.032, 1.056). Similarly, in the validation set (*p* = 0.00016, HR = 1.122, 95% CI: 1.070, 1.175), the Kaplan-Meier survival analysis in conjunction with the log-rank test underscored the substantial prognostic value of the radiomics model in effectively stratifying patients into high and low-risk groups.

### Development and validation of the clinical nomogram

After conducting a univariate Cox regression, we found that four clinical risk factors were significant: odynuria, previous malignancies, pathological grading, and MIS (*p* < 0.05). Finally, by multivariate Cox regression analysis, these four risk factors were incorporated, leading to the identification of odynuria and previous malignancies as independent risk factors within the clinical model, both with *p* values of less than 0.05 (refer to Table 2). The C-index for the clinical model was determined to be 0.611 (95% CI, 0.579–0.643) within the training set and 0.583 (95% CI, 0.536–0.630) within the validation set.

**Table 2:** Cox univariate and multivariate proportional hazard models of risk factors for recurrence of BCa

Characteristics	Univariate		Multivariate	
	HR (95% CI)	*p*	HR (95% CI)	*p*
Age (years)	1.019 (0.994–1.044)	0.136		
BMI	0.985 (0.923–1.051)	0.639		
Gender	0.696 (0.330–1.468)	0.341		
Smoking	0.908 (0.538–1.532)	0.718		
Drinking	0.961 (0.518–1.783)	0.900		
Frequent urination	1.523 (0.873–2.658)	0.138		
Urinary urgency	1.417 (0.812–2.472)	0.220		
Odynuria	2.355 (1.358–4.086)	0.002	2.214 (1.190–4.117)	0.012
Urinary incontinence	1.108 (0.443–2.772)	0.827		
Low back pain	0.330 (0.080–1.353)	0.123		
Total cholesterol	1.034 (0.829–1.291)	0.765		
Triglyceride	1.265 (0.997–1.606)	0.053		
High-density lipoprotein	1.163 (0.465–2.906)	0.747		
Low-density lipoprotein	1.040 (0.770–1.404)	0.798		
Urea	0.989 (0.842–1.162)	0.894		
Creatinine	1.002 (0.984–1.020)	0.827		
Uric acid	1.000 (0.997–1.004)	0.784		
Previous malignancies	3.192 (1.508–6.757)	0.002	4.327 (1.987–9.420)	< 0.001
Tumor location	1.165 (0.801–1.695)	0.423		
Tumor size	1.018 (0.592–1.749)	0.950		
Tumor number	1.096 (0.628–1.912)	0.748		
Pathological grading	1.890 (1.106–3.230)	0.020	1.369 (0.637–2.940)	0.421
MIS	1.954 (1.167–3.273)	0.011	1.136 (0.502–2.568)	0.760
Infusion drug	1.012 (0.781–1.312)	0.928		
Surgical methods	0.847 (0.596–1.204)	0.355		

*HR* Hazard ratio, *CI* Confidence interval

### Development and validation of the radiomics-clinical nomogram

We constructed a radiomics-clinical nomogram by incorporating the independent risk factors from the clinical model with the radscore derived from the radiomics model. This final model consisted of three key predictors: odynuria, previous malignancies, and the radscore. Utilizing these predictors, we developed a comprehensive nomogram capable of predicting the 1-year, 2-year, and 3-year recurrence probabilities for BCa patients.

**Fig. 5:**
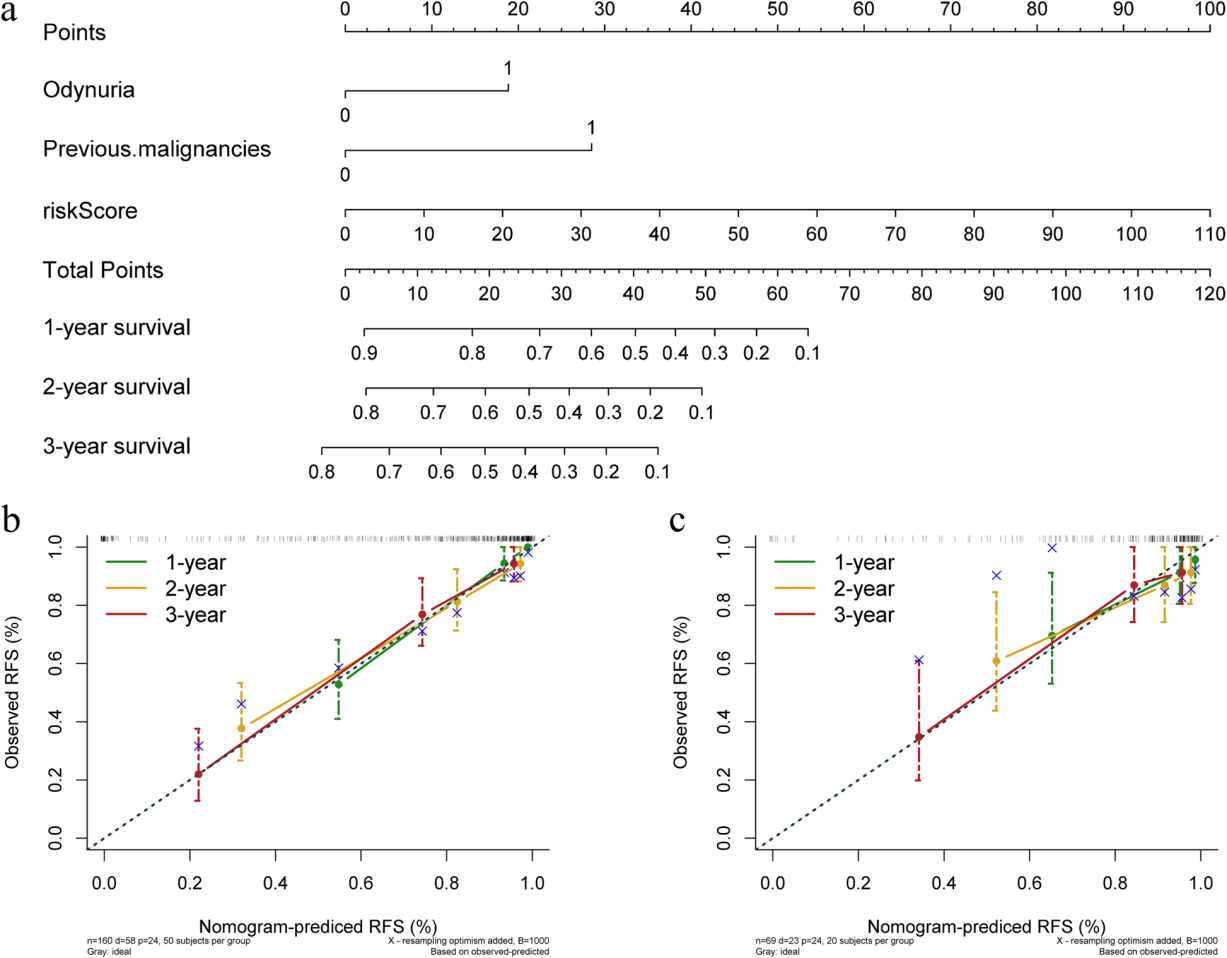
The nomogram integrates odynuria, previous malignancies and radscore to predict 1-year, 2-year, and 3-year RFS of BCa (**a**). The calibration curve evaluates the agreement between the predicted RFS probability of the nomogram and the actual RFS probability in the training set (**b**) and validation set (**c**)

The combined model exhibited a notable C-index of 0.853 (95% CI, 0.829–0.877) within the training set and 0.832 (95% CI, 0.784–0.880) within the validation set, surpassing the prognostic performance of the radiomics model when considered in isolation. The radiomics-clinical nomogram incorporating these three independent predictors is visually depicted in Fig. 5a. Additionally, calibration plots for both the training and validation sets are presented in Fig. 5b and c.

**Fig. 6:**
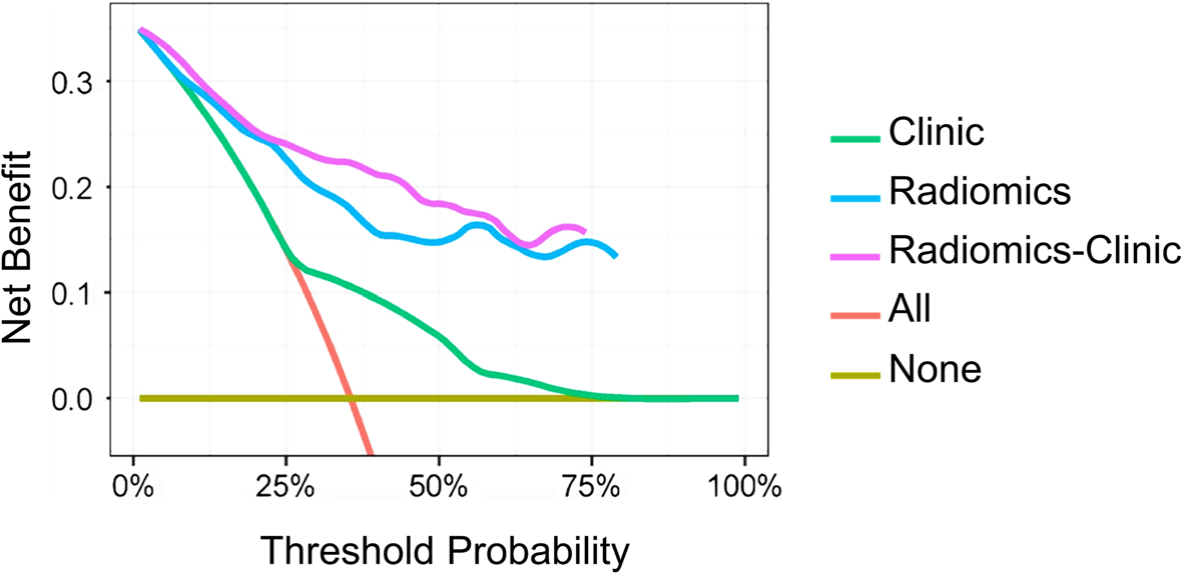
Decision curve analysis for the clinical model, radiomics model, and combined model for BCa recurrence risk assessment. Note: the *Y*-axis represents the net benefit; the *X*-axis represents the threshold probability. The net clinical benefit of the radiomics-clinical model was higher than that of the radiomics and clinical model across all risk threshold probabilities and higher than that of the two reference cases, namely the red line of “All” (all patients considered as recurrence) or the yellow line of “None” (all patients considered as no recurrence)

To gauge the predictive advantages of the radiomics-clinical nomogram compared to the clinical model or radiomics model, we utilized NRI for assessing the 3-year RFS. The NRI for the radiomics-clinical nomogram model, in comparison to the clinical model, was determined to be 0.6768 (95% CI: 0.5549–0.7987, *p* < 0.001). The result shows that the radiomics-clinical nomogram improves the efficiency of BCa recurrence risk stratification by 67.68% compared to the clinical model alone. When compared to the radiomics model, the NRI for the radiomics-clinical nomogram was 0.0233 (95% CI: 0.0720–0.1187, *p* = 0.631), indicating a modest 2.33% increase in diagnostic efficacy for the combined model over the radiomics model. Figure 6 shows that the radiomics-clinical model outperforms the radiomics and clinical models alone, providing superior net benefits within a wide range of threshold probabilities.

## Discussion

The high recurrence rate stands as a pivotal factor significantly impacting prognosis of BCa patients. Accurately predicting recurrence risk is crucial for tailoring treatment strategies and follow-up plans. In the 2021 European Association of Urology (EAU) score model [26], high-risk NMIBC patients have been shown to still have a high recurrence rate after TURBT and are prone to progression to MIBC [27]. RC combined with PLND is currently the standard treatment for patients with MIBC [28]. However, some patients may refuse RC because of the potential complications associated with this procedure including massive blood loss, risk of infection, paralytic ileus, and potential decreased quality of life. To address this clinical challenge, urologists are actively exploring a precise recurrence risk stratification aiming to provide an alternative to bladder protection strategies for individuals who are unwilling or unsuitable for surgery [29, 30]. At present, cystoscopy is the gold standard for cancer tracking of urinary bladder, but due to the inherent heterogeneity of tumor lesions, it may lead to potential misdiagnosis and thus delay in treatment [31].

Radiomics is a bridge between medical images and computable data [32]. This study explored a nomogram to evaluate the recurrence risk of BCa, aiming to predict the RFS of BCa patients undergoing surgical treatment and guide future clinical management. Our radiological characteristics encompassed 22 distinct features, comprising three ADC sequences, four DWI sequences, seven DCE sequences, and eight T2WI sequences. Through internal validation, we determined that the radscore derived from multi-sequence MRI imaging was an independent predictor associated with clinical risk factors (*p* < 0.001). This finding underscores the advantages of a multi-sequence MRI-radiomics model in effectively stratifying BCa recurrence risk. It also highlights that different MRI sequences can complement and augment each other in the realm of imaging diagnosis and prognosis evaluation for BCa, aligning with prior research in this area. Two studies by Wang et al. [19, 33] compared postoperative DCE and DWI information in BCa patients and found that the DWI sequence was better than the DCE sequence in differentiating postoperative BCa recurrence and inflammation in patients. Additionally, the addition of DWI to enhanced mucosal basement DCE could improve tumor detection and enhance the imaging staging diagnosis of BCa.

Prior investigations have shown that specific factors, including age, gender, histological grade, MIS, tumor size, the number of tumors, and the choice of surgical intervention, wield significant influence over the recurrence of BCa [3, 34, 35]. In our study, we conducted a comprehensive analysis to assess the factors influencing RFS in BCa. The results of our univariate Cox regression analysis highlighted the significance of odynuria, previous malignancies, pathological grading, and MIS in relation to RFS in BCa patients (*p* < 0.05). These findings align with previous research in this field.

To further gauge the value of incorporating clinical risk features into our predictive model, we identified two clinically independent predictors, namely odynuria and previous malignancies, in addition to the radscore. Notably, the two clinical risk factors had not been extensively studied among BCa recurrence factors. However, our nomogram suggested their pivotal role in BCa recurrence risk. Our results unveiled that the combined nomogram outperformed both the radiomics model (0.853 vs. 0.823) and the clinical model alone (0.853 vs. 0.611) in terms of C-index, demonstrating its superior predictive capability. This trend was similarly observed in the validation set (0.832 vs. 0.811 vs. 0.583). Similarly, DCA analysis underscored the nomogram’s superiority over the radiomics model or clinical model within a wide range of risk thresholds, providing a greater net benefit.

Currently, radiomics methods have emerged as a popular solution for the clinical challenges faced in preoperative prediction of MIS and tumor grade in BCa. However, there is a lack of research on the effectiveness of radiomics features in predicting tumor recurrence. It is worth noting that our nomogram is not the first to study radiomics in predicting the prognosis of BCa. In 2019, Xu et al. [36] developed and validated a personalized prediction model for the first 2 years of recurrence risk based on multi-sequence MRI radiomics. Their model achieved good results with an accuracy of 88% and an AUC of 0.915 in the training set and an accuracy of 80.95% and an AUC of 0.838 in the validation set. Zhang et al. [25] established a lasso model that included a single DWI sequence, combined with the radscore, clinical pathology, and other factors, achieving good performance in predicting individual PFS. However, these studies had incomplete scanning sequences and the study size was small, which required further external validation. Based on the current relevant researches, our study highlighted the advantages of using multi-sequence MRI in soft tissue tumor diagnosis and multi-level information presentation, incorporating clinical risk factors to develop a combined nomogram as a powerful tool for personalized RFS prediction in BCa patients. Compared with traditional clinical models, this model showed strong promise and was guaranteed to be thoroughly validated in future research efforts.

Our research had certain limitations that need to be addressed. Firstly, the patient cohort was limited to a single institution, and retrospective studies may have some inherent bias. Secondly, the study did not include relevant genomic information. Key genomic biomarkers such as Ki67, FGFR3, and p53, which are strongly associated with RFS, should be incorporated into the nomogram to enhance its predictive performance [37, 38]. Furthermore, the advancement of deep learning research has demonstrated promising results in predicting BCa recurrence and assessing efficacy [12, 39]. While its clinical validation is pending, we hold the expectation that radiology can substantially enhance the efficiency of recurrence prediction. Our future endeavors involve the integration of a substantial dataset encompassing multi-center and multi-parametric MRI data for leveraging deep learning technology in lesion delineation and feature data analysis. Furthermore, we intend to incorporate additional critical prognostic indicators, such as genomic features, to further augment the predictive capabilities of the nomogram. The validation of this extended model will be carried out using multi-center data, advancing its reliability and applicability.

## Conclusions

To sum up, our study suggests that multi-sequence MRI-based radiomics features hold promise as a valuable tool for assessing the recurrence risk of BCa patients. Additionally, the integration of radiomics with clinical risk factors into a nomogram may have the potential to enhance individualized predictions for BCa patients’ RFS. However, it is important to note that further validation of this approach is warranted.

## Data Availability

The datasets used and/or analyzed during the current study are available from the corresponding author on reasonable request.

## Abbreviations

BCa: Bladder cancer
CI: Confidence interval
DCA: Decision curve analysis
HR: Hazard ratio
ICC: Intraclass correlation coefficient
LASSO: Least absolute shrinkage and selection operator
MIBC: Muscle-invasive bladder cancer
MIS: Myometrial invasion status
NMIBC: Non-muscle-invasive bladder cancer
NRI: Net reclassification improvement
PFS: Progression-free survival
PLND: Pelvic lymph node dissection
RC: Radical cystectomy
RFS: Recurrence-free survival
TURBT: Transurethral resection of bladder tumor
VOI: Volume of interest
